# Lymphocyte density determined by computational pathology validated as a predictor of response to neoadjuvant chemotherapy in breast cancer: secondary analysis of the ARTemis trial

**DOI:** 10.1093/annonc/mdx266

**Published:** 2017-05-19

**Authors:** H. R. Ali, A. Dariush, J. Thomas, E. Provenzano, J. Dunn, L. Hiller, A.-L. Vallier, J. Abraham, T. Piper, J. M. S. Bartlett, D. A. Cameron, L. Hayward, J. D. Brenton, P. D. P. Pharoah, M. J. Irwin, N. A. Walton, H. M. Earl, C. Caldas

**Affiliations:** 1 Li Ka Shing Centre, Cancer Research UK Cambridge Institute;; 2 Department of Pathology;; 3 Institute of Astronomy, University of Cambridge, Cambridge;; 4 Edinburgh Cancer Research Centre, Western General Hospital, Edinburgh;; 5 Department of Oncology, University of Cambridge, Addenbrooke’s Hospital, Cambridge;; 6 Department of Histopathology, Addenbrooke’s Hospital, Cambridge University Hospitals NHS Foundation Trust, Cambridge;; 7 Cambridge Experimental Cancer Medicine Centre and NIHR Cambridge Biomedical Research Centre, Cambridge;; 8 Warwick Clinical Trials Unit, University of Warwick, Coventry, UK;; 9 Ontario Institute for Cancer Research, Toronto, Canada

**Keywords:** tumour-infiltrating lymphocytes, neoadjuvant chemotherapy, breast cancer, predictive, computational pathology

## Abstract

**Background:**

We have previously shown lymphocyte density, measured using computational pathology, is associated with pathological complete response (pCR) in breast cancer. The clinical validity of this finding in independent studies, among patients receiving different chemotherapy, is unknown.

**Patients and methods:**

The ARTemis trial randomly assigned 800 women with early stage breast cancer between May 2009 and January 2013 to three cycles of docetaxel, followed by three cycles of fluorouracil, epirubicin and cyclophosphamide once every 21 days with or without four cycles of bevacizumab. The primary endpoint was pCR (absence of invasive cancer in the breast and lymph nodes). We quantified lymphocyte density within haematoxylin and eosin (H&E) whole slide images using our previously described computational pathology approach: for every detected lymphocyte the average distance to the nearest 50 lymphocytes was calculated and the density derived from this statistic. We analyzed both pre-treatment biopsies and post-treatment surgical samples of the tumour bed.

**Results:**

Of the 781 patients originally included in the primary endpoint analysis of the trial, 609 (78%) were included for baseline lymphocyte density analyses and a subset of 383 (49% of 781) for analyses of change in lymphocyte density. The main reason for loss of patients was the availability of digitized whole slide images. Pre-treatment lymphocyte density modelled as a continuous variable was associated with pCR on univariate analysis (odds ratio [OR], 2.92; 95% CI, 1.78–4.85; *P* < 0.001) and after adjustment for clinical covariates (OR, 2.13; 95% CI, 1.24–3.67; *P* = 0.006). Increased pre- to post-treatment lymphocyte density showed an independent inverse association with pCR (adjusted OR, 0.1; 95% CI, 0.033–0.31; *P* < 0.001).

**Conclusions:**

Lymphocyte density in pre-treatment biopsies was validated as an independent predictor of pCR in breast cancer. Computational pathology is emerging as a viable and objective means of identifying predictive biomarkers for cancer patients.

**ClinicalTrials.gov:**

NCT01093235.

Key Message

We show that fully automated computational pathology can accurately determine lymphocyte density in digital slides of pre- and post-treatment breast tumours. Using the ARTemis randomized trial, we validate our previous observations that higher lymphocyte density is associated with pCR and that a paradoxical increase in pre- to post-treatment lymphocyte density is associated with residual disease.

## Introduction

Tumour-infiltrating lymphocytes (TILs) have been widely investigated as a prognostic and predictive biomarker in breast cancer [1]. However, routine assessment of TILs in the clinical setting is hindered by poor reproducibility of their manual pathological evaluation. We previously conducted a systematic analysis of quantitative pathology metrics in the Neo-tAnGo trial [2] and found that pre-treatment tumour lymphocyte density was independently associated with pathological complete response (pCR) [3]. Our observations suggest that computational pathology performs as well as pathologist read scores. Moreover, it is automated, objective and quantitative and may, therefore, facilitate clinical implementation. In addition, we found that a relative increase in lymphocyte density after treatment was inversely associated with pCR and that this relationship significantly differed by taxane sequencing [3], suggesting that in a subset of patients chemotherapy modulates the post-treatment immune microenvironment.

The ARTemis trial showed that the addition of bevacizumab to standard neoadjuvant chemotherapy significantly increased the proportion of patients with a pCR [4], but this has not impacted on disease-free and overall survival [5]. Here, we tested whether our original findings could be validated in this independent study, and have also conducted exploratory analyses of associations with disease-free and overall survival.

## Methods

### Study design

ARTemis was a multicentre phase III randomized controlled trial conducted to test whether the addition of bevacizumab to three cycles of docetaxel, followed by three cycles of fluorouracil, epirubicin and cyclophosphamide increased the proportion of patients with a pCR [4]. Women with human epidermal growth factor receptor 2 (HER2)-negative early breast cancer were recruited from May 2009 until January 2013. Of the 800 patients randomized, 781 were available for the primary endpoint analysis. The primary endpoint was pCR (absence of invasive cancer in both the breast and lymph nodes). Here, whether a pCR had occurred was either determined based on central pathology review or, where central review was not possible, on histopathology reports [6]. Details of eligibility and follow-up procedures are provided in the main trial report [4]. The trial was approved by the multicentre and local research ethics committees. All patients provided written, informed consent. The trial was registered at ClinicalTrials.gov (NCT01093235). Supplementary Table S1, available at *Annals of Oncology* online, details characteristics of patients included in this analysis.

### Computational pathology

Digital whole slide images of haematoxylin and eosin (H&E) stained tissue sections both before and after treatment, were captured using a Hamamatsu Nanozoomer (Hamamatsu City, Shizuoka Pref., Japan). Blinded to all pathological and clinical parameters, we used our computational pathology analysis pipeline to compute cellular metrics from these images. Supplementary Figure S1, available at *Annals of Oncology* online, summarizes the computational pathology workflow. Briefly, the algorithm segments cell nuclei and, based on a training set of approximately 1000 objects per category, uses machine learning (support-vector-machine) to classify cells into three categories: cancer, stromal and lymphocyte. Finally, based on these classes descriptive cellular metrics are computed, including cellular density. Here lymphocyte density is calculated as follows: for every detected lymphocyte in a section, the average distance R to the 50 nearest lymphocytes (*N *=* *50) is calculated using a K-nearest-neighbour approach.

For each lymphocyte, density is estimated as *N*/(π*R*^2^) and the median of this value for all detected lymphocytes is taken as the summary statistic for a given section. The computational pathology approach has been described in detail previously [3] and the analysis code is available at http://www.ast.cam.ac.uk/∼adariush/files/codes/.

### Statistical analyses

We tested for associations between lymphocyte density and pCR using logistic regression, reporting odds ratios (OR) and 95% confidence intervals (95% CI). Lymphocyte density and change in lymphocyte density were modelled as continuous variables. Multivariable models were adjusted for age, randomization arm, histological grade, estrogen receptor (ER) status, tumour size and lymph node status at randomization. Age and histological grade were modelled as continuous variables. Tumour size (<51 mm versus >50 mm) and lymph node status (negative versus positive) were modelled as categorical variables. Associations with categorical clinical variables were tested using Kruskal–Wallis tests. Associations with overall survival (OS) defined as all-cause mortality, and disease-free survival (DFS) were tested using Cox proportional-hazards models, where follow-up commenced from day of surgery. DFS was calculated to date of first relapse (loco-regional or distant, not including DCIS); to date of death in women dying without invasive relapse; or to date of censoring in women alive and disease free. Survival analyses were conducted separately by ER-status to account for known violations of the proportional-hazards assumption [7]. Statistical analyses were conducted using Stata SE version 14.2 (Stata Corp, College Station, TX).

## Results

**Figure 1.**
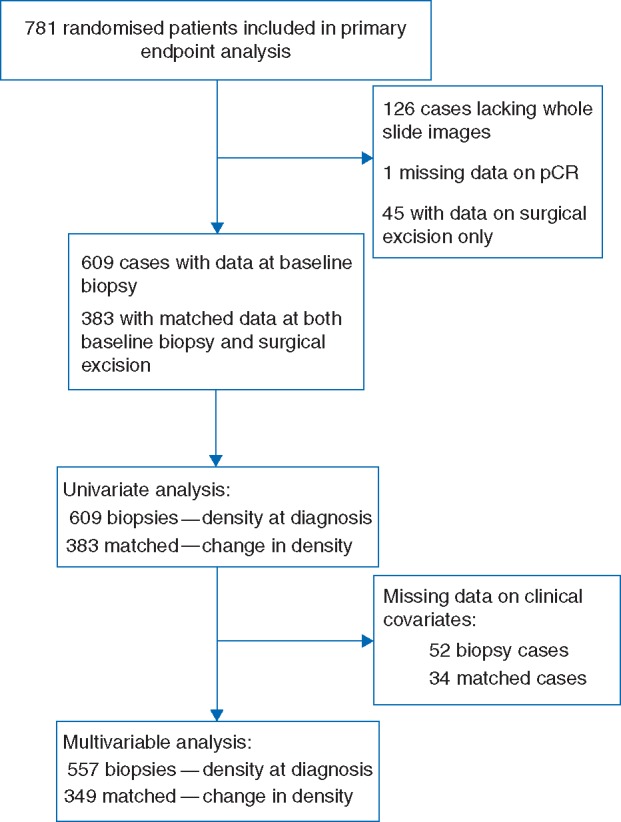
Flowchart of patients and samples through analytic stages.

Of the 781 patients included in the ARTemis primary analysis, 609 (78%) had computational pathology and baseline outcome data (Figure 1), where 109 (18%) experienced pCR, a similar proportion to the entire group of 781 patients where 20% experienced pCR. Of these 609, 383 patients had matched pre- and post-treatment samples to calculate change in lymphocyte density; of which 17 (4%) achieved pCR (supplementary Table S1, available at *Annals of Oncology* online). Median time at risk for OS was 3.1 years (range 0.07–6.3 years). Among the 609 patients, there were 140 DFS events and 98 OS events.

Pre-treatment lymphocyte density was associated with ER status (*P *<* *0.001), tumour size (*P *=* *0.003), and histological grade (*P *<* *0.001) (supplementary Figure S2, available at *Annals of Oncology* online).

**Figure 2.**
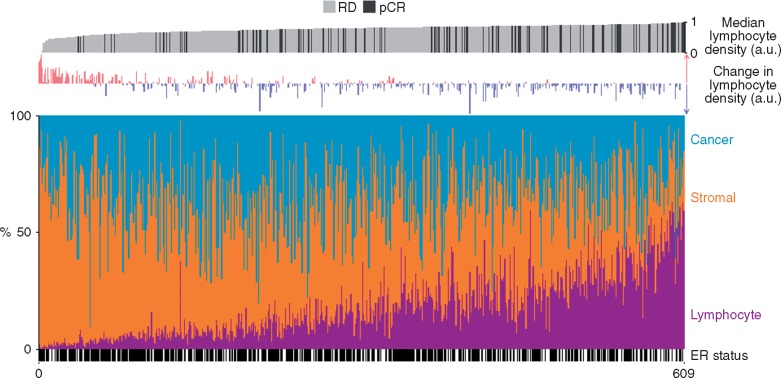
Association between lymphocyte density, change in lymphocyte density, cellular proportions and chemotherapy response. Observations are ranked by pre-treatment lymphocyte density scores. Lymphocyte density has been rescaled to between zero and one for illustration. a.u., arbitrary units; pCR, pathological complete response; RD, residual disease.

Higher pre-treatment lymphocyte density was associated with a greater chance of pCR in unadjusted (OR, 2.93; 95% CI, 1.77–4.85; *P *<* *0.001) and adjusted (OR, 2.13; 95% CI, 1.24–3.67; *P *=* *0.006) analyses (Table 1 and Figure 2). However, there was no association between pre-treatment lymphocyte density and survival (OS or DFS) in either ER-positive or ER-negative disease (supplementary Table S2, available at *Annals of Oncology* online). Consistent with our previous observations [3], an increase in lymphocyte density between pre- and post-treatment was associated with residual disease (adjusted OR for pCR, 0.1; 95% CI, 0.033–0.31; *P *<* *0.001; Figure 2 and supplementary Table S3, available at *Annals of Oncology* online). Change in lymphocyte density was not associated with OS or DFS in either ER-positive or ER-negative disease (supplementary Table S2, available at *Annals of Oncology* online).

**Table 1:** Univariable and multivariable logistic regression of lymphocyte density and clinical covariates against pCR

Variable	Categories	Univariate				Multivariate			
		Odds ratio	95% CI	*P* value	Observations	Odds ratio	95% CI	*P* value	Observations
Median lymphocyte density	Continuous	2.93	1.77–4.85	0.00003	609	2.13	1.24–3.67	0.006	557
Grade	1,2,3	4.82	2.80–8.29	<0.00001	557	2.80	1.58–4.96	0.0004	
ER status	Negative, Positive	0.19	0.12–0.30	<0.00001	609	0.29	0.18–0.47	<0.00001	
Age	Continuous	0.97	0.94–0.99	0.007	609	0.98	0.95–1.00	0.06	
Node status	Negative, Positive	0.69	0.45–1.04	0.08	609	0.65	0.41–1.05	0.08	
Chemotherapy	BEV+D FEC, D FEC	0.72	0.48–1.10	0.13	609	0.60	0.38–0.97	0.04	
Tumour size	<51 mm, >50 mm	0.73	0.42–1.26	0.25	609	1.05	0.56–1.97	0.87	

a.u., arbitrary units, FEC, fluorouracil, epirubicin and cyclophosphamide; BEV, bevacizumab; pCR, pathological complete response.

## Discussion

In this computational pathology analysis of the ARTemis trial, we have validated our previous observation that higher pre-treatment lymphocyte density is associated with pCR and that an increase in lymphocyte density after treatment is seen in a subset of surgical resection samples with residual disease.

Pre-treatment lymphocyte density, while predicting pCR independent of clinical variables, was not associated with survival. Although this contrasts with the findings of past studies [8–10], it should be noted that in these published reports lymphocyte density was not quantified using the approach described here. Our finding should also be interpreted cautiously since analyses were modestly powered due to small sample sizes and limited follow-up time.

Our analyses were limited to tissue morphology in H&E slides. While this is a pragmatic and therefore clinically feasible approach, it overlooks functional differences in infiltrating lymphocytes, which have been shown to influence clinical outcome [11–13]. A second limitation was the incomplete representation of post-treatment specimens. A possible explanation for this, and for the lower proportion of patients with pCR in this subset, is that slides from surgical samples in which a pCR is observed are less likely to be digitized since they do not contain cancer cells. Similarly, we were not able to include all patients recruited to the trial because some slides were not available for digitization. Importantly, the findings validate those of our previous independent study and therefore are more likely to be generalizable.

Our findings validate pre-treatment lymphocyte density—a computational pathology metric—as a predictor of pCR. This highlights that automated quantitative pathology can perform at a level comparable to pathologist-read scores and may therefore improve the standard histopathological evaluation of tumour samples. Such approaches have the additional advantage of being objective and reproducible. Moreover, our finding that an increase in pre- to post-treatment lymphocyte density is associated with residual disease again highlights perturbations in the immune microenvironment secondary to, and presumably caused by, treatment. We speculate that such a comparative metric could serve as a biomarker to identify patients likely to respond to post-neoadjuvant immunotherapy.

Higher pre-treatment lymphocyte density is validated as a predictor of pCR among women with early stage breast cancer. In addition, an increase in lymphocyte density following chemotherapy is again observed to be associated with residual disease. Patients with low pre-treatment lymphocyte density may benefit from more aggressive therapies or enrolment into clinical trials. In addition, immunotherapies may prove more effective following an increase in lymphocyte density following neoadjuvant chemotherapy. 

## Funding

Cancer Research UK (CRUK/08/037), Roche, Sanofi-Aventis. 

## Disclosure

The authors have declared no conflicts of interest.

## Supplementary Material

Supplementary Table S1

Supplementary Table S2

Supplementary Table S3

Supplementary Figure 1

Supplementary Figure 2
