# Supplementary material for: Lymphocyte density determined by computational pathology validated as a predictor of response to neoadjuvant chemotherapy in breast cancer: secondary analysis of the ARTemis trial
Source: Ann Oncol. 2017 May 19;28(8):1832–5. doi: 10.1093/annonc/mdx266 (PMC5834010; doi:10.1093/annonc/mdx266)
Supplement: Supplementary Table S1 [file supplementary_table1_mdx266.docx]

Supplementary Table 1. Summary of patient characteristics.

|  | Pathological complete response | | | | | |
| --- | --- | --- | --- | --- | --- | --- |
|  | **No pCR** | | **pCR** | | **Total** | |
|  | ***N*** | **%** | ***N*** | **%** | ***N*** | **%** |
| Tumour size |  |  |  |  |  |  |
| <51mm | 393 | 78.6 | 91 | 83.5 | 484 | 79.5 |
| >50mm | 107 | 21.4 | 18 | 16.5 | 125 | 20.5 |
| Total | 500 | 100 | 109 | 100 | 609 | 100 |
| Node status |  |  |  |  |  |  |
| Negative | 233 | 46.6 | 61 | 56 | 294 | 48.3 |
| Positive | 267 | 53.4 | 48 | 44 | 315 | 51.7 |
| Total | 500 | 100 | 109 | 100 | 609 | 100 |
| Grade |  |  |  |  |  |  |
| 1 | 12 | 2.4 | 1 | 0.9 | 13 | 2.1 |
| 2 | 212 | 42.4 | 15 | 13.8 | 227 | 37.3 |
| 3 | 229 | 45.8 | 88 | 80.7 | 317 | 52.1 |
| Missing | 47 | 9.4 | 5 | 4.6 | 52 | 8.5 |
| Total | 500 | 100 | 109 | 100 | 609 | 100 |
| Chemotherapy |  |  |  |  |  |  |
| BEV+D FEC | 235 | 47 | 60 | 55 | 295 | 48.4 |
| D FEC | 265 | 53 | 49 | 45 | 314 | 51.6 |
| Total | 500 | 100 | 109 | 100 | 609 | 100 |
| ER status |  |  |  |  |  |  |
| Negative | 117 | 23.4 | 67 | 61.5 | 184 | 30.2 |
| Positive | 383 | 76.6 | 42 | 38.5 | 425 | 69.8 |
| Total | 500 | 100 | 109 | 100 | 609 | 100 |
| Diagnostic biopsies |  |  |  |  |  |  |
| Analysed | 500 | 100 | 109 | 100 | 609 | 100 |
| Total | 500 | 100 | 109 | 100 | 609 | 100 |
| Surgical samples |  |  |  |  |  |  |
| Missing | 134 | 26.8 | 92 | 84.4 | 226 | 37.1 |
| Analysed | 366 | 73.2 | 17 | 15.6 | 383 | 62.9 |
| Total | 500 | 100 | 109 | 100 | 609 | 100 |
| All-cause mortality |  |  |  |  |  |  |
| No | 417 | 83.4 | 93 | 85.3 | 510 | 83.7 |
| Yes | 83 | 16.6 | 16 | 14.7 | 99 | 16.3 |
| Total | 500 | 100 | 109 | 100 | 609 | 100 |
| Disease recurrence |  |  |  |  |  |  |
| No | 378 | 75.6 | 89 | 81.7 | 467 | 76.7 |
| Yes | 122 | 24.4 | 20 | 18.3 | 142 | 23.3 |
| Total | 500 | 100 | 109 | 100 | 609 | 100 |
|  | **No pCR** | | | **pCR** | | |
|  | ***N*** | **Median** | **IQR** | ***N*** | **Median** | **IQR** |
| Median lymphocyte density^*^ | 500 | -4.324 | -4.755 to -4.065 | 109 | -4.145 | -4.482 to -3.910 |
| Change in lymphocyte density^†^ | 366 | -0.154 | -0.431 to 0.185 | 17 | -0.473 | -1.207 to -0.228 |

^*^Log-transformed values

^†^Pre-post treatment value

Abbreviations: FEC, fluorouracil, epirubicin and cyclophosphamide; BEV, bevacizumab; pCR, pathological complete response
