# Supplementary material for: Lymphocyte density determined by computational pathology validated as a predictor of response to neoadjuvant chemotherapy in breast cancer: secondary analysis of the ARTemis trial
Source: Ann Oncol. 2017 May 19;28(8):1832–5. doi: 10.1093/annonc/mdx266 (PMC5834010; doi:10.1093/annonc/mdx266)
Supplement: Supplementary Table S2 [file supplementary_table2_mdx266.docx]

Supplementary Table 2. Univariate Cox proportional hazards models for disease-free and overall survival, separately for ER-positive and ER-negative disease

|  |  | **ER-positive** | | | | | **ER-negative** | | | | |
| --- | --- | --- | --- | --- | --- | --- | --- | --- | --- | --- | --- |
| **Variable** | **Categories** | **HR** | **95% CI** | ***P*-value** | **Observations** | **Events** | **HR** | **95% CI** | ***P*-value** | **Observations** | **Events** |
| **Overall survival** | | | | | | | | | | | |
| Median lymphocyte density^*^ | Continuous | 1.34 | 0.79-2.28 | 0.28 | 418 | 49 | 1.02 | 0.58-1.78 | 0.95 | 178 | 49 |
| Change in lymphocyte density^*^ | Continuous | 0.68 | 0.39-1.21 | 0.19 | 277 | 31 | 1.12 | 0.69-1.82 | 0.64 | 97 | 34 |
| **Disease-free survival** | | | | | | | | | | | |
| Median lymphocyte density^*^ | Continuous | 1.27 | 0.85-1.92 | 0.24 | 418 | 80 | 0.92 | 0.55-1.52 | 0.74 | 178 | 60 |
| Change in lymphocyte density^*^ | Continuous | 0.73 | 0.47-1.14 | 0.17 | 277 | 50 | 1.34 | 0.84-2.16 | 0.22 | 97 | 38 |

^*^Arbitrary units
