# Supplementary material for: Lymphocyte density determined by computational pathology validated as a predictor of response to neoadjuvant chemotherapy in breast cancer: secondary analysis of the ARTemis trial
Source: Ann Oncol. 2017 May 19;28(8):1832–5. doi: 10.1093/annonc/mdx266 (PMC5834010; doi:10.1093/annonc/mdx266)
Supplement: Supplementary Table S3 [file supplementary_table3_mdx266.docx]

Supplementary Table 3. Univariate and multivariate logistic regression analyses for change in lymphocyte density and clinical covariates against pCR.

|  |  | **Univariate** | | | | **Multivariate** | | | |
| --- | --- | --- | --- | --- | --- | --- | --- | --- | --- |
| **Variable** | **Categories** | **Odds ratio** | **95% CI** | ***P*-value** | **Observations** | **Odds ratio** | **95% CI** | ***P*-value** | **Observations** |
| Median lymphocyte density^*^ | Continuous | 2.93 | 1.77-4.85 | 0.00003 | 609 | 0.49 | 0.093-2.61 | 0.41 | 349 |
| Change in lymphocyte density^*^ | Continuous | 0.24 | 0.11-0.50 | 0.0001 | 383 | 0.10 | 0.033-0.31 | 0.00007 |  |
| Age | Continuous | 0.97 | 0.94-0.99 | 0.007 | 609 | 1.03 | 0.96-1.10 | 0.44 |  |
| Tumour size | <51mm, >50mm | 0.73 | 0.42-1.26 | 0.25 | 609 | 0.70 | 0.13-3.69 | 0.67 |  |
| Grade | 1,2,3 | 4.82 | 2.80-8.29 | <0.00001 | 557 | 6.06 | 1.16-31.7 | 0.03 |  |
| ER status | Negative, Positive | 0.19 | 0.12-0.30 | <0.00001 | 609 | 0.14 | 0.031-0.59 | 0.008 |  |
| Chemotherapy | BEV+D FEC, D FEC | 0.72 | 0.48-1.10 | 0.13 | 609 | 4.49 | 1.01-20.0 | 0.05 |  |
| Node status | Negative, Positive | 0.69 | 0.45-1.04 | 0.08 | 609 | 0.69 | 0.20-2.36 | 0.56 |  |

^*^Arbitrary units

Abbreviations: FEC, fluorouracil, epirubicin and cyclophosphamide; BEV, bevacizumab; pCR, pathological complete response
