# Supplementary figures and images for: Lymphocyte density determined by computational pathology validated as a predictor of response to neoadjuvant chemotherapy in breast cancer: secondary analysis of the ARTemis trial
Source: Ann Oncol. 2017 May 19;28(8):1832–5. doi: 10.1093/annonc/mdx266 (PMC5834010; doi:10.1093/annonc/mdx266)

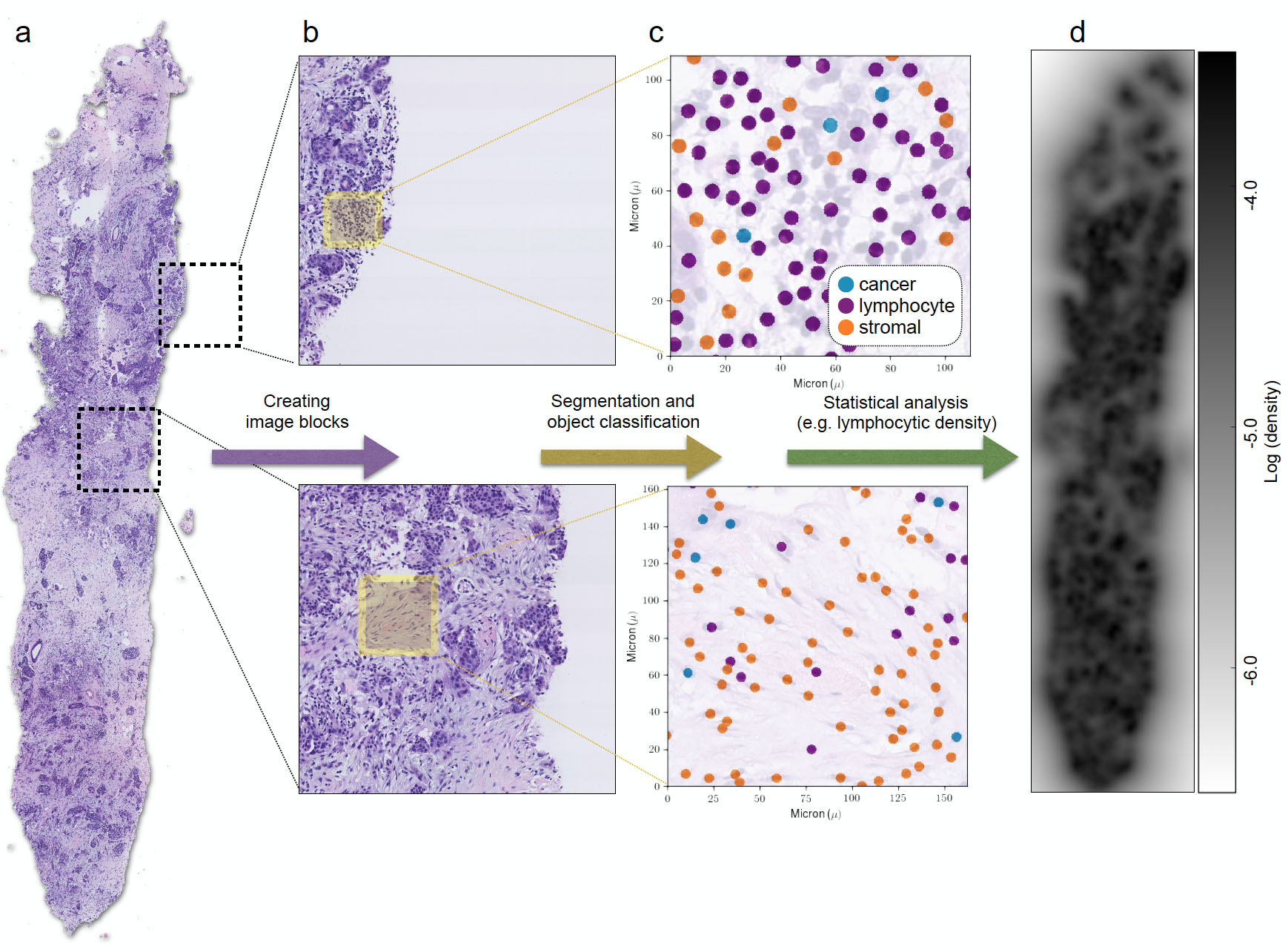

Supplement: Supplementary Figure 1 [file figure4paper_mdx266.jpeg]

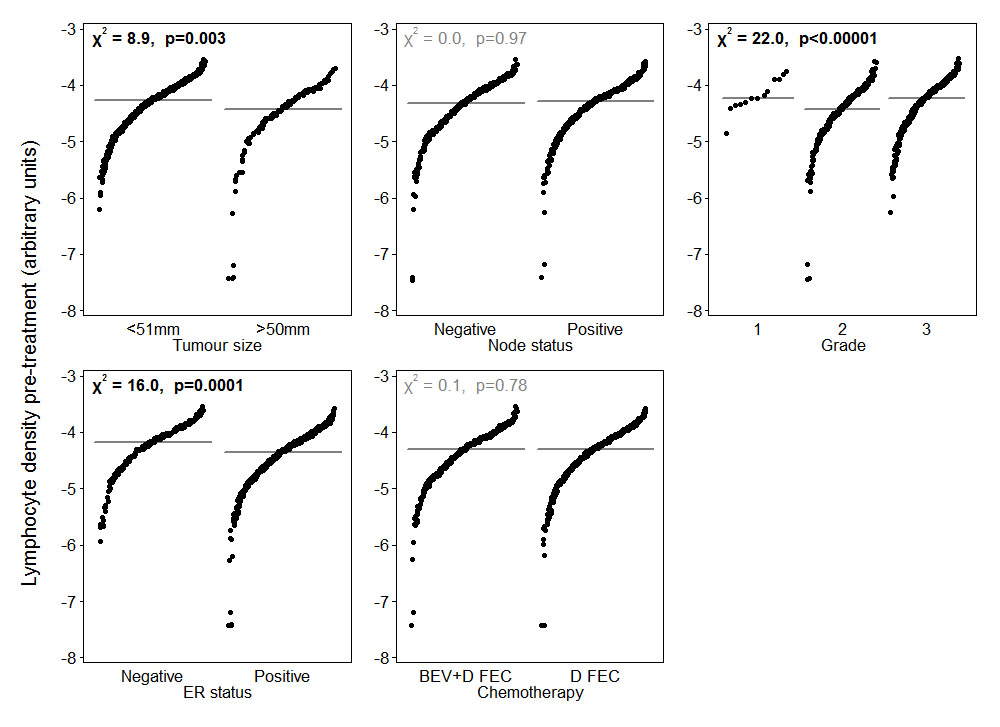

Supplement: Supplementary Figure 2 [file lym_d50_by_clinical_mdx266.jpeg]
